# Supplementary material for: Investigating centrifugal filtration of serum-based FTIR spectroscopy for the stratification of brain tumours
Source: PLoS One. 2023 Feb 17;18(2):e0279669. doi: 10.1371/journal.pone.0279669 (PMC9937474; doi:10.1371/journal.pone.0279669)
Supplement: S1 File — (DOCX) [file pone.0279669.s001.docx]

**Investigating centrifugal filtration of serum-based FTIR spectroscopy for the stratification of brain tumours**

SUPPORTING INFORMATION


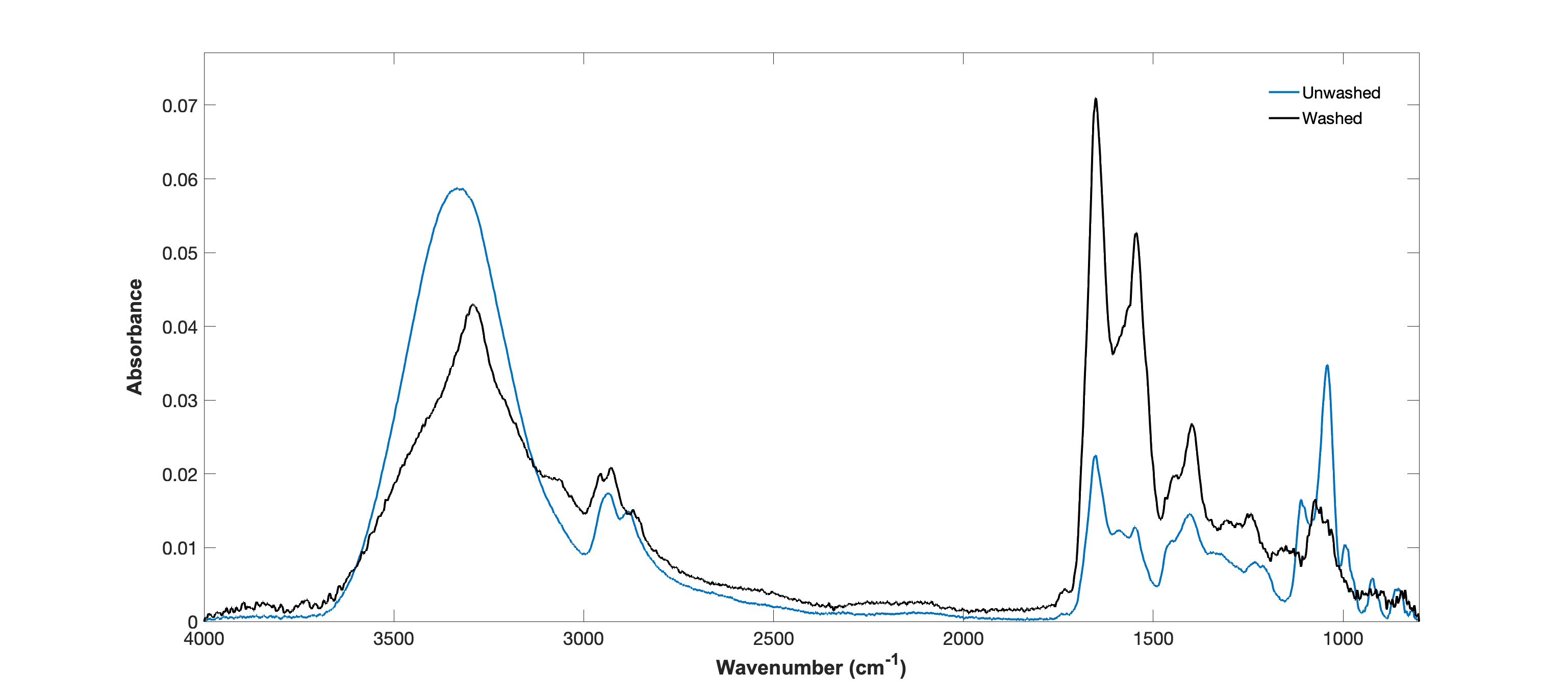


**Example spectra of a non-cancer** **patient.**

Serum was fractionated through two 100 kDa molecular weight cut-off filters. The blue line represents the spectral profile with an unwashed filter and the black line is a washed filter.

**Patient serum spectra including unfiltered whole serum and each molecular weight region included.**

Average of the 30 lymphoma patients shown here. The inset is the wavenumber region between 1800 cm^-1^ and 1000 cm^-1^, which was used for all chemometrics and machine learning analyses. Spectra is offset for clearer visualisation.

**Patient serum spectra including unfiltered whole serum and each molecular weight region included.**

Average of the 30 non-cancer patients shown here. The inset is the wavenumber region between 1800 cm^-1^ and 1000 cm^-1^, which was used for all chemometrics and machine learning analyses. Spectra is offset for clearer visualisation.


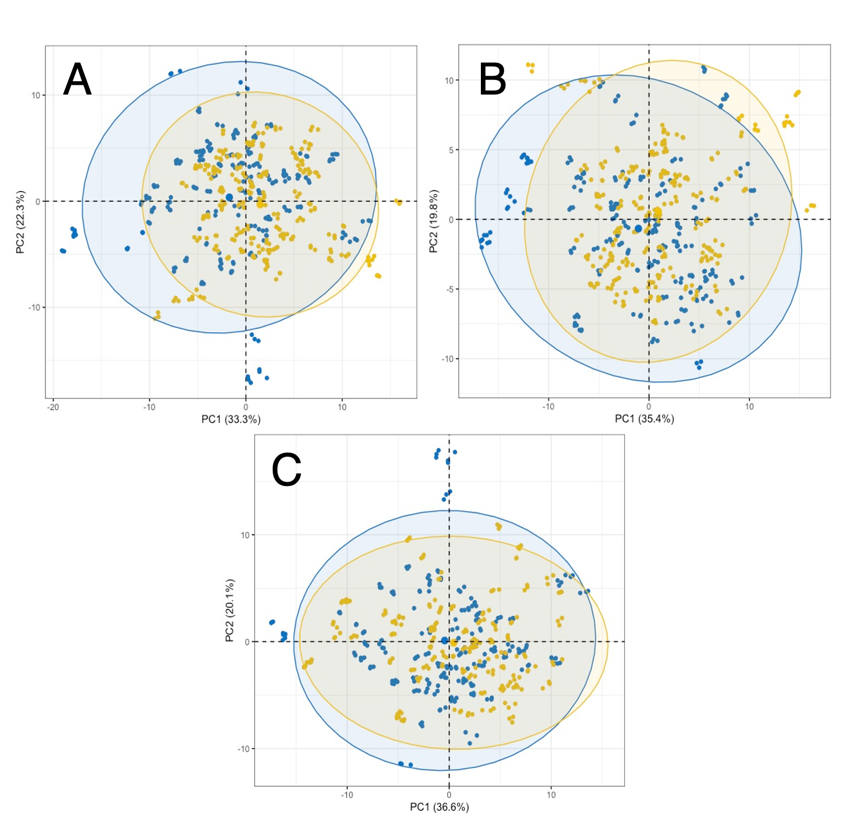


**PCA scores plots for the filtered serum (<50 kDa) of the first and second dimensions.**

The three figures represent (A) GBM in blue and non-cancer in yellow, (B) Lymphoma in blue and non-cancer in yellow and (C) GBM in blue and lymphoma in yellow. The eclipses in each class represent a 95% confidence interval. Values in parentheses are the TEV for each PC.


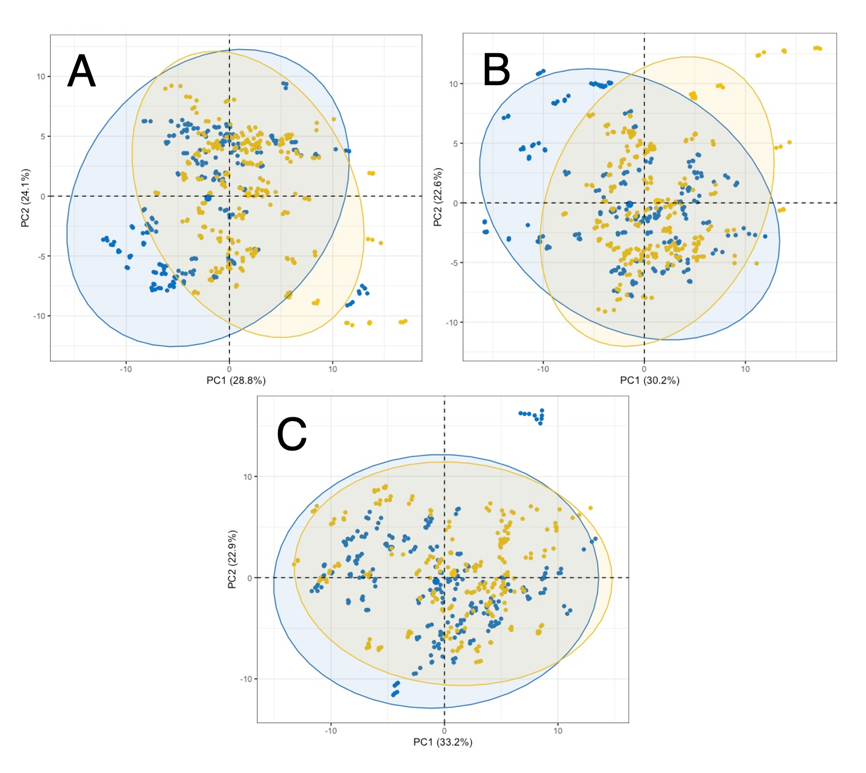


**PCA scores plots for the filtered serum (<30 kDa) of the first and second dimensions.**

The three figures represent (A) GBM in blue and non-cancer in yellow, (B) Lymphoma in blue and non-cancer in yellow and (C) GBM in blue and lymphoma in yellow. The eclipses in each class represent a 95% confidence interval. Values in parentheses are the TEV for each PC.


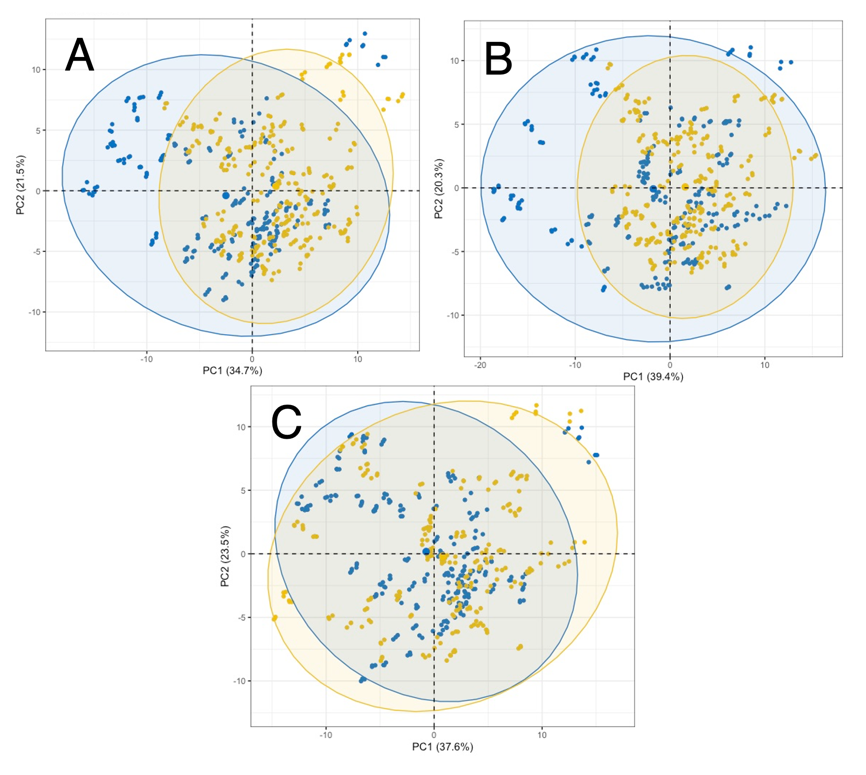


**PCA scores plots for the filtered serum (<10 kDa) of the first and second dimensions.**

The three figures represent (A) GBM in blue and non-cancer in yellow, (B) Lymphoma in blue and non-cancer in yellow and (C) GBM in blue and lymphoma in yellow. The eclipses in each class represent a 95% confidence interval. Values in parentheses are the TEV for each PC.


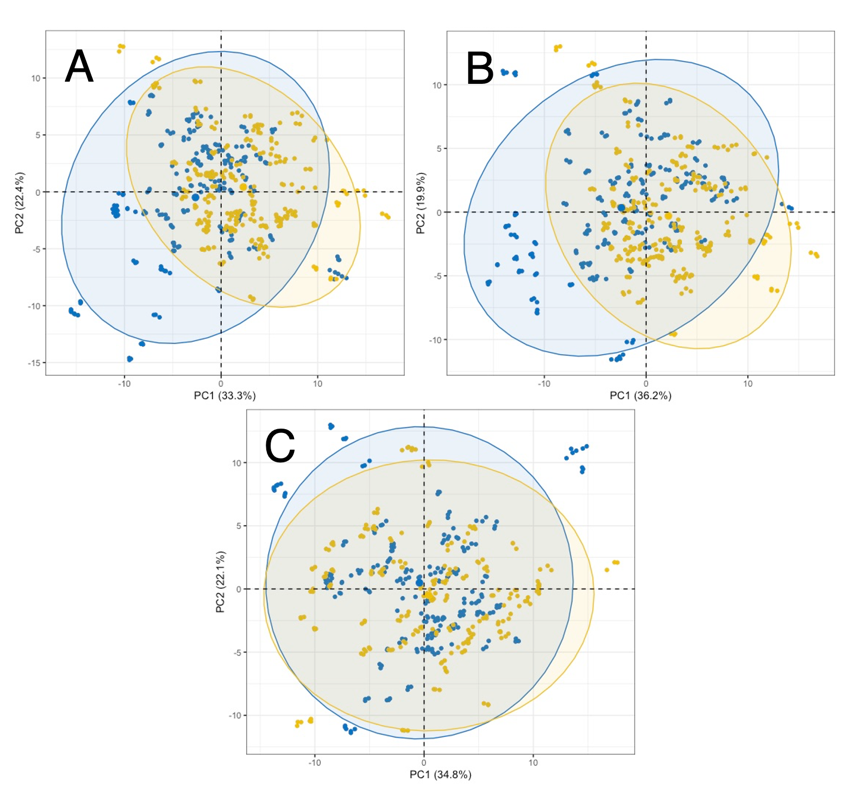


**PCA scores plots for the filtered serum (<3 kDa) of the first and second dimensions.**

The three figures represent (A) GBM in blue and non-cancer in yellow, (B) Lymphoma in blue and non-cancer in yellow and (C) GBM in blue and lymphoma in yellow. The eclipses in each class represent a 95% confidence interval. Values in parentheses are the TEV for each PC.

**Sensitivity, specificity and balanced accuracies for the RF model classification of GBM *versus* non-cancer patients. Mean, standard deviation (SD) and 95% confidence intervals (CIs) are provided.**

|  |  | **Sensitivity (%)** | | | **Specificity (%)** | | | **Balanced accuracy (%)** | | |
| --- | --- | --- | --- | --- | --- | --- | --- | --- | --- | --- |
|  |  | Mean | SD | 95% CI | Mean | SD | 95% CI | Mean | SD | 95% CI |
| GBM  *versus*  NC | Unfiltered | 88.9 | 9.7 | ±2.7  86.2-91.6 | 94.8 | 6.8 | ±1.9  92.9-96.7 | 91.8 | 6.0 | ±1.6  90.2-93.4 |
|  | <100 kDa | 85.0 | 14.1 | ±3.9  81.1-88.9 | 81.7 | 12.1 | ±3.3  78.4-85.0 | 83.4 | 9.5 | ±2.6  80.8-86.0 |
|  | <50 kDa | 73.4 | 16.9 | ±4.6  68.8-78.0 | 74.3 | 15.9 | ±4.4  69.9-78.7 | 73.8 | 10.6 | ±2.9  70.9-76.7 |
|  | <30 kDa | 76.2 | 17.3 | ±4.7  71.5-80.9 | 76.3 | 15.1 | ±4.1  72.2-80.4 | 76.2 | 10.0 | ±2.7  73.5-78.9 |
|  | <10 kDa | 69.6 | 17.9 | ±4.9  64.7-74.5 | 81.5 | 14.7 | ±4.0  77.5-85.5 | 75.5 | 9.3 | ±2.6  72.9-78.1 |
|  | <3 kDa | 78.7 | 18.6 | ±5.1  73.6-83.8 | 81.0 | 14.8 | ±4.1  76.9-85.1 | 79.9 | 9.2 | ±2.5  77.4-82.4 |

**Sensitivity, specificity and balanced accuracies for the RF model classification of lymphoma *versus* non-cancer patients. Mean, standard deviation (SD) and 95% confidence intervals (CIs) are provided.**

|  |  | **Sensitivity (%)** | | | **Specificity (%)** | | | **Balanced accuracy (%)** | | |
| --- | --- | --- | --- | --- | --- | --- | --- | --- | --- | --- |
|  |  | Mean | SD | 95% CI | Mean | SD | 95% CI | Mean | SD | 95% CI |
| Lymphoma  *versus*  NC | Unfiltered | 83.8 | 13.8 | ±3.8  80.0-87.6 | 94.3 | 7.8 | ±2.1  92.2-96.4 | 89.1 | 6.9 | ±1.9  87.2-91.0 |
|  | <100 kDa | 59.1 | 20.4 | ±5.6  53.5-64.7 | 73.4 | 14.9 | ±4.1  69.3-77.5 | 66.3 | 11.6 | ±3.2  63.1-69.5 |
|  | <50 kDa | 54.3 | 17.8 | ±4.9  49.4-59.2 | 61.7 | 14.1 | ±3.9  57.8-65.6 | 58.0 | 11.1 | ±3.0  55.0-61.0 |
|  | <30 kDa | 59.4 | 18.2 | ±5.0  54.4-64.4 | 72.8 | 13.2 | ±3.6  69.2-76.4 | 66.1 | 9.7 | ±2.7  63.4-68.8 |
|  | <10 kDa | 58.6 | 16.5 | ±4.5  54.1-63.1 | 70.2 | 14.1 | ±3.9  66.3-74.1 | 64.4 | 10.1 | ±2.8  61.6-67.2 |
|  | <3 kDa | 67.2 | 17.8 | ±4.9  62.3-72.1 | 62.5 | 19.4 | ±5.3  57.2-67.8 | 64.8 | 9.2 | ±2.5  62.3-67.3 |

**Sensitivity, specificity and balanced accuracies for the RF model classification of GBM *versus* Lymphoma patients. Mean, standard deviation (SD) and 95% confidence intervals (CIs) are provided.**

|  |  | **Sensitivity (%)** | | | **Specificity (%)** | | | **Balanced accuracy (%)** | | |
| --- | --- | --- | --- | --- | --- | --- | --- | --- | --- | --- |
|  |  | Mean | SD | 95% CI | Mean | SD | 95% CI | Mean | SD | 95% CI |
| GBM  *versus*  lymphoma | Unfiltered | 86.3 | 10.4 | ±2.9  83.4-89.2 | 84.1 | 13.7 | ±3.8  80.3-87.9 | 85.2 | 8.1 | ±2.2  83.0-87.4 |
|  | <100 kDa | 50.4 | 22.6 | ±6.2  44.2-56.6 | 53.5 | 19.3 | ±5.3  48.2-58.8 | 52.0 | 13.6 | ±3.7  48.3-55.7 |
|  | <50 kDa | 49.6 | 20.5 | ±5.6  44.0-55.2 | 36.4 | 20.7 | ±5.7  30.7-42.1 | 43.0 | 13.4 | ±3.7  39.3-46.7 |
|  | <30 kDa | 57.8 | 20.8 | ±5.7  52.1-63.5 | 45.7 | 20.8 | ±5.7  40.0-51.4 | 51.8 | 12.1 | ±3.3  48.5-55.1 |
|  | <10 kDa | 38.5 | 14.3 | ±3.9  34.6-42.4 | 47.9 | 15.1 | ±4.1  43.8-52.0 | 43.2 | 9.4 | ±2.6  40.6-45.8 |
|  | <3 kDa | 50.0 | 18.0 | ±4.9  45.1-54.9 | 39.2 | 19.6 | ±5.4  33.8-44.6 | 44.6 | 11.5 | ±3.2  41.4-47.8 |


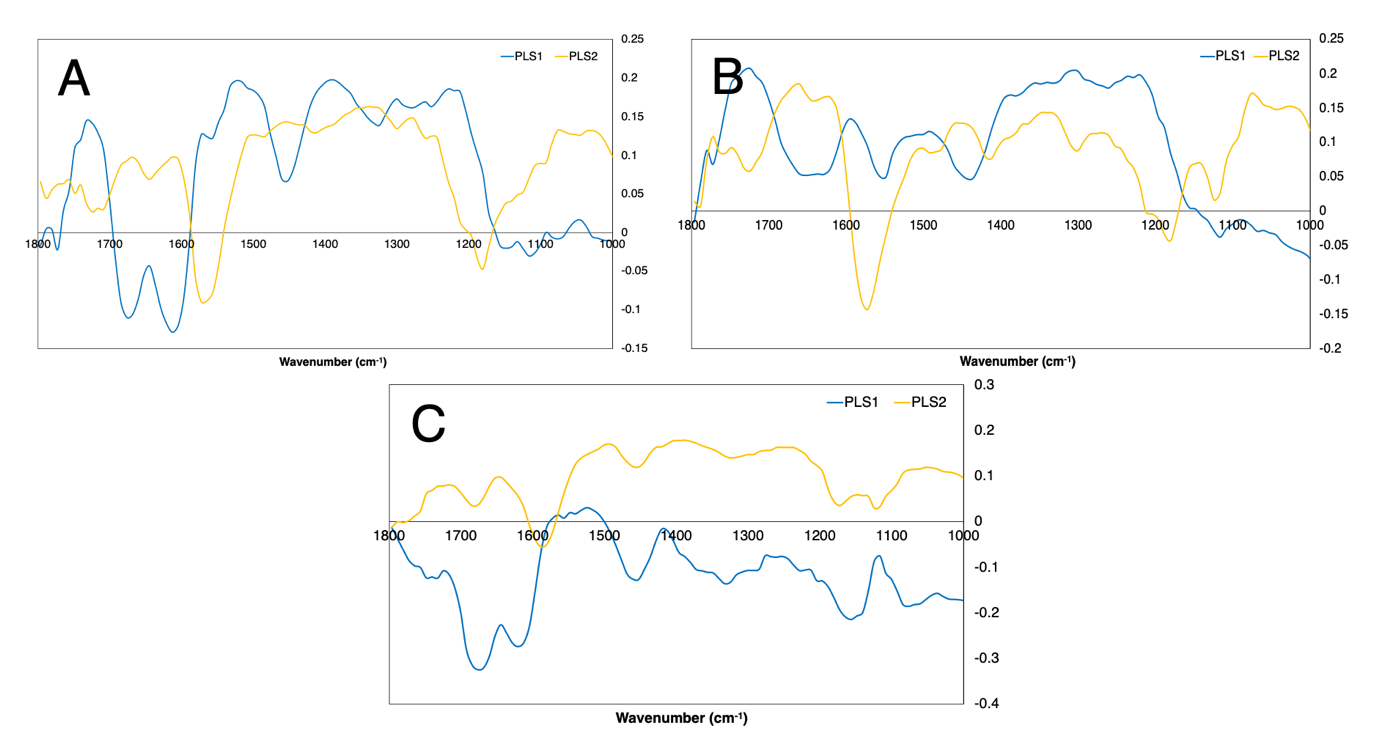


**PLS loadings plot for the 1st and 2nd LVs for the filtered serum (<50 kDa).**

(A) GBM *versus* non-cancer, (B) Lymphoma *versus* non-cancer and (C) GBM *versus* lymphoma.


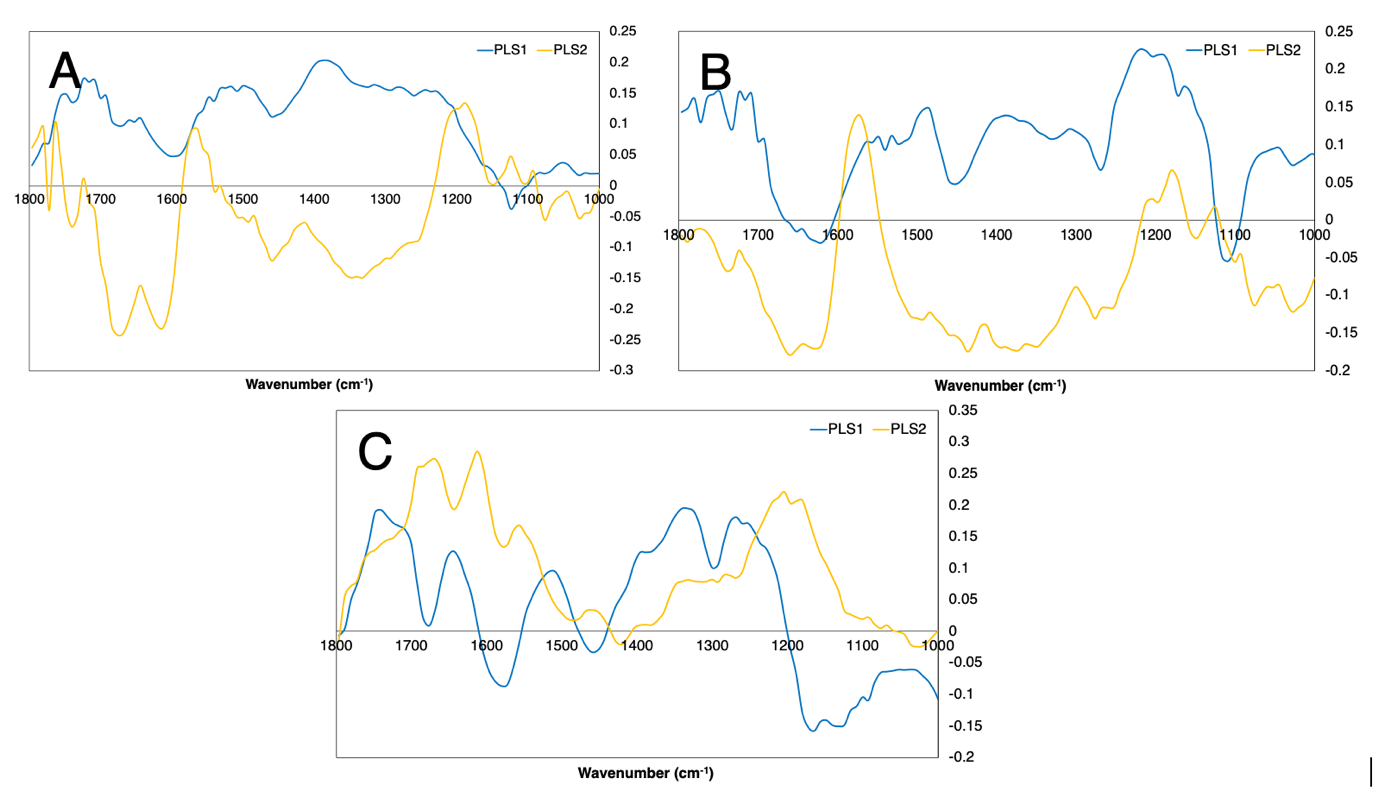


**PLS loadings plot for the 1st and 2nd LVs for the filtered serum (<30 kDa).**

(A) GBM *versus* non-cancer, (B) Lymphoma *versus* non-cancer and (C) GBM *versus* lymphoma.


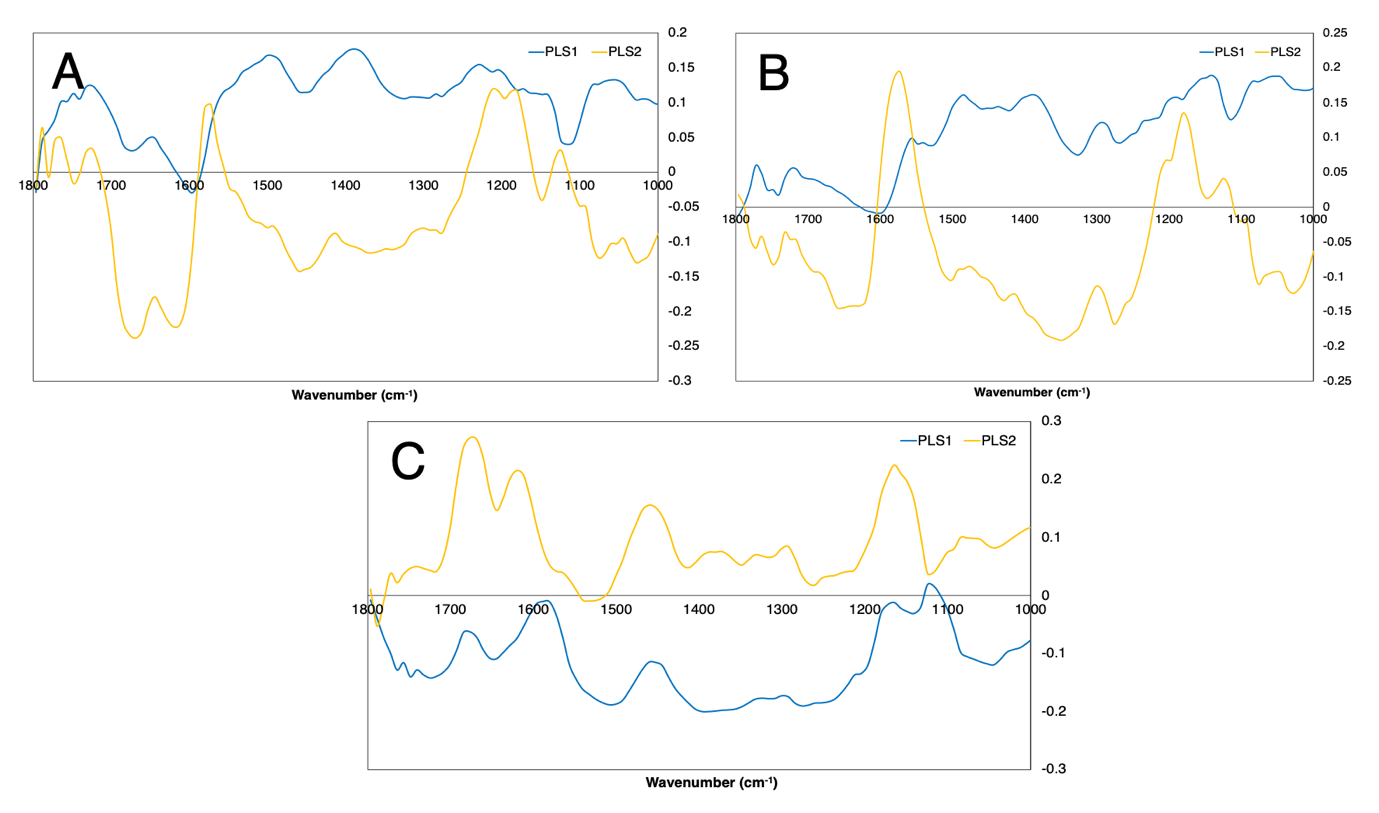


**PLS loadings plot for the 1st and 2nd LVs for the filtered serum (<10 kDa).**

(A) GBM *versus* non-cancer, (B) Lymphoma *versus* non-cancer and (C) GBM *versus* lymphoma.


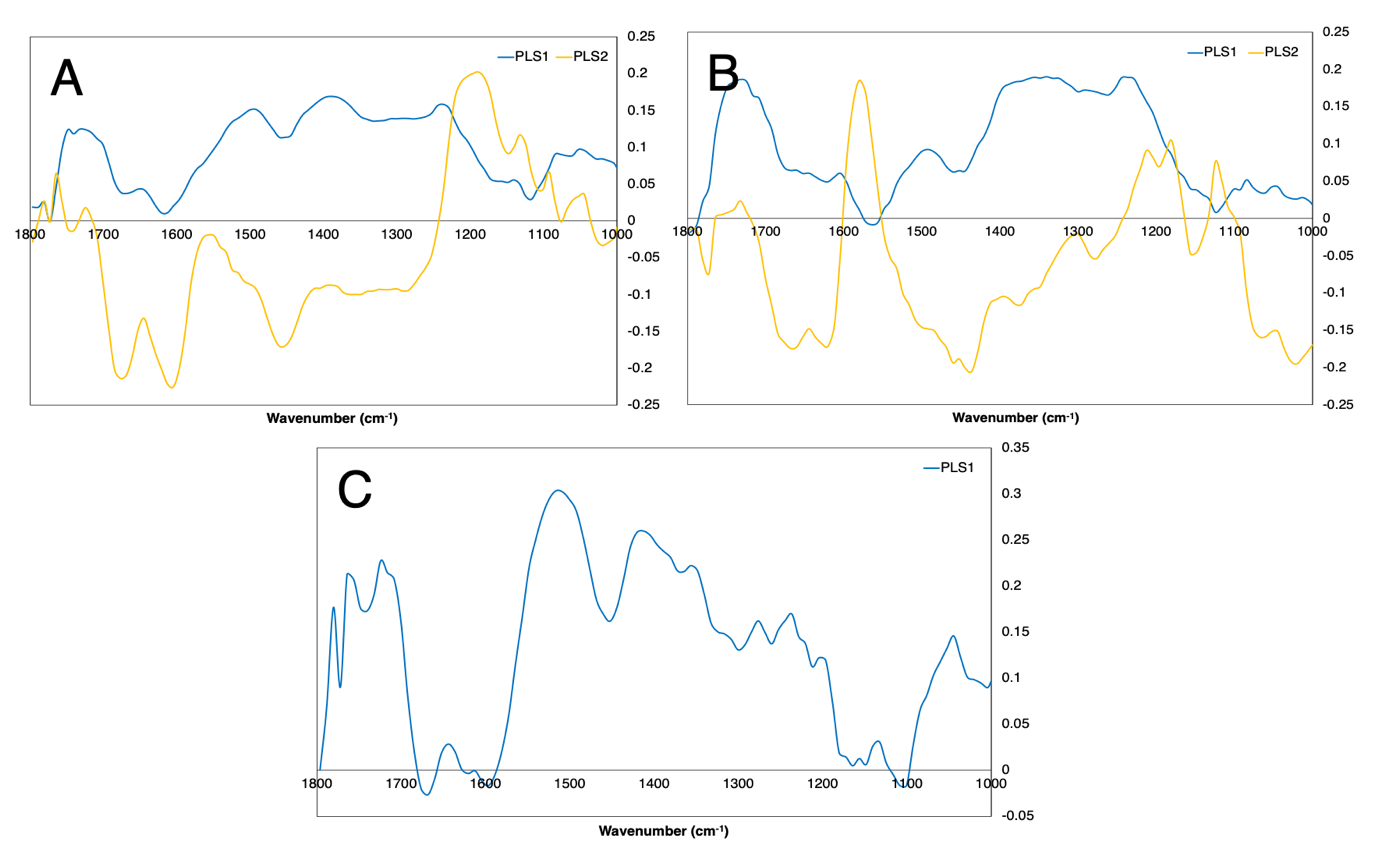


**PLS loadings plot for the 1st and 2nd LVs for the filtered serum (<3 kDa**).

(A) GBM *versus* non-cancer, (B) Lymphoma *versus* non-cancer and (C) GBM *versus* lymphoma.
